# A comparative analysis of self-identification and functional measures of disability^☆^

**DOI:** 10.1016/j.dhjo.2025.101980

**Published:** 2025-11-06

**Authors:** Stephanie Rennane, Zachary A. Morris

**Affiliations:** a RAND, 1200 S. Hayes Street, Arlington, VA, 22206, United States; b Stony Brook University, United States

**Keywords:** Disability policy, Self-identification, Survey measurement, Functioning

## Abstract

**Background:**

Disability is typically measured in surveys using functional limitation questions rather than asking respondents to self-identify as having a disability. Little is known about the characteristics of those who self-identify with a disability and how they compare with those identified via functional limitation questions.

**Objective:**

To compare the prevalence and characteristics of people with disabilities measured by both functional and self-identification measures, and to assess the overlap between these populations.

**Methods:**

Using nationally representative survey data from 2023, we conduct bivariate comparisons of demographic, health, functional characteristics and financial needs between populations captured by self-identification question, the Washington Group Short Set on Functioning (WG-SS) and the American Community Survey Six (ACS-6) Questions. We estimate a multivariable regression to explore predictors of self-identification.

**Results:**

Adding a self-identification question doubles the population with disabilities relative to measuring disability with the WG-SS alone, and increases the population by 30 % relative to the ACS-6 alone. People who self identify are less likely to be female or Hispanic/Latinx, are in worse physical health and more likely to be LGBTQIA + compared to those identified only by functional measures. The group identified by both the self-identification and either functioning question set are in the worst health and are more likely to participate in disability programs.

**Conclusion:**

Including a self-identification question in addition to functioning questions expands the prevalence of disability to varying degrees depending on which functional questions are used, but use of functioning and self-identification questions together enhances identification of the subgroup with highest needs.

## 1. Introduction

The World Health Organization International Classification of Functioning, Disability and Health (ICF) defines disability as “an umbrella term for impairments, activity limitations and participation restrictions”.^1^ Disability is not static: it can be chronic or episodic; temporary or permanent, and involves a consideration of personal identity.^2^ The ICF recognizes the importance of environmental factors, implying that activity limitations or restrictions may vary across settings. For example, an individual with limited mobility may fully participate on a Zoom call but be excluded from settings with stairs. A person with a hearing or intellectual impairment, on the other hand, may experience the reverse. All this complexity makes disability an extremely difficult concept to measure comprehensively and cohesively.

Accurate measurement of the population of people with disabilities is important for many reasons, including counting the size of this population for representation, identifying inequities, and identifying populations for service delivery. Measurement of this population also has potential implications for disaster planning and funding allocations for many public programs.^3^ The population with disabilities, along with most other subgroups, is most commonly measured through nationally representative surveys. Yet the challenges in defining the concept of disability present challenges for survey measurement as well. On surveys, disability is usually measured through functional limitation questions rather than by directly asking respondents about disability status or identity. Two main question batteries measure disability: the American Community Survey-Six Question module (ACS-6) and the Washington Group Short Set (WG-SS). Both measures ask six questions about functioning and classify a respondent as having a disability if they indicate difficulties with any one of the six functional domains. Five domains overlap across the two metrics: seeing, hearing, walking, remembering and self-care. The final ACS-6 question asks about difficulty running errands, while the WG-SS asks about difficulty communicating. The primary difference between the ACS-6 and the WG-SS concerns how they measure difficulty. In the ACS-6, individuals are classified as having a disability if they answer “yes” to any of the questions. Individuals are classified as having a disability if they “cannot do” or have “a lot of difficulty” with (rather than no or some difficulty) any of the WG-SS questions. See Weeks et al., 2021 for more discussion of the WG-SS and ACS-6.^3^

Prior research has identified the limitations of functional measures in capturing the full population of people with disabilities.^4–6^ Among other issues, these six-question functional measures lack questions about psychological impairments^c^ and ask respondents to assess their functional status at the current time, which may not be an accurate measure for respondents with intermittent, accommodated or episodic limitations.^7^ Further, chronic conditions including Long COVID may not directly limit functioning in ways covered by these questions, which could exclude more respondents.^7^ A measure of self-identified disability could provide an opportunity to capture these populations. However, a self-identified measure is more subjective and could introduce misalignment between those who report functional limitations but do not consider themselves disabled.^8^ In this paper, we compare the populations identified with a disability by functional questions and a question asking whether respondents self-identify with a disability.

Several studies have examined the cohesion of functional measures such as the ACS-6 and WG-SS.^9–11^ Other work has debated the potential value of combining the ACS-6 with a question of self-reported work--limiting disability in identifying recipients of disability benefits (SSI/SSDI).^12,13^ Few prior studies have compared the cohesion of functional measures with a question of general self-identification of disability and to our knowledge, no prior study compares the WG-SS, ACS and the self-identification of disability within a nationally representative sample. Hall et al., 2022 and Hall et al., 2024 assess the share of individuals identified by both the WG-SS and ACS-6 *within* a population that self-identifies as people with disabilities.^7,14^ Salinger et al., 2023 compare the ACS-6 and self-identified populations in a nationally representative sample and, in accordance with Hall, find misalignment between the two groups.^8^

This paper advances the literature in two ways. First, drawing on a nationally representative survey of the US adult (18+) population, we provide novel data on the population of people with disabilities who self-identify and compare this group with both the ACS and WG-SS measures. Second, we provide new analyses about the health and use of disability-related goods and services of each population subgroup. We thus examine the extent to which functional and self-identification measures, both singularly and in combination, identify the population of people with disabilities in the poorest health and with the highest support needs.

## 2. Data and methods

We analyze the Survey Disability-related Goods and Services (SDGS), which we fielded to a nationally representative online panel, the Understanding America Study (UAS), in June 2023. UAS panel members answer researchers’ queries once to twice a month via an online interface.

The SDGS included a disability screener administered to all panel members. A total of 9088 respondents answered the SDGS screener. The disability screener included the Washington Group Short Set (WG-SS) questions and asked all respondents: “Do you consider yourself to be a person with a disability?” See Morris et al., 2025 for more details.^15^ If the respondent self-identified, they were also asked to characterize their disability (e.g., physical, learning, mental or psychological, vision, hearing, intellectual, developmental, or other).

An advantage of the UAS is that responses to other surveys can be matched to the same panel respondents. In April 2023, the Financial Health Network fielded the Financial Health Pulse Survey, including the ACS-6 questions, to panelists.^16,17^ We link responses from the Financial Health Pulse Survey to SDGS screener respondents to analyze a nationally representative sample of the population who was asked the ACS-6, WG-SS and self-identification questions. The match rate between the two surveys was 81 %, yielding a total sample size of 7950. Our analysis focuses on the population of individuals ages 18–69 who were measured as having a disability based on meeting the criteria of either WG-SS or ACS-6, or who responded “yes” to the self identification question. In total, 1411 respondents met these criteria. UAS survey methodologists adjusted the survey weights to the matched sample. To our knowledge, this is the only dataset that exists containing all three question measures for comparison in the general adult U.S. population. We provide a consort diagram describing the full sample selection process in Fig. S1.

We characterize the size of the population with a disability based on the WG-SS, ACS-6 and those screened under only the self-identification question or the functional questions. We conduct bivariate comparisons of demographic, health and functional characteristics between these groups. In addition to the WG-SS and ACS-6 functional measures, we compare age, race/ethnicity, gender, marital status, sexual orientation, household income, education levels, employment status, disability program participation, self-reported health and mental health, the PHQ-4 anxiety and depression screening tool, and self-reported rates of pain. Finally, we examine respondents’ expenditures and need for disability-related goods from the second part of the SDGS survey, including total out of pocket expenditures on disability-related goods, any unmet-need for disability-related goods, and any unmet-need for disability-related goods in ten specific functional domains. We do not have this information for respondents from the Financial Health Survey if they only identified with a disability based on the ACS-6 and were not asked the second part of the SDGS survey. Therefore, we compare need and use of disability related goods only between the WG-SS and self-identified populations.

We also conducted multivariable regressions to examine which factors were most predictive of an individual self-identifying with a disability. In this analysis, our primary outcome variable was an indicator for whether the person answered “yes” to the self-identification question. We included the demographic and health characteristics described above in the model. We included individuals who either self-identified or were identified as having a disability based on either ACS-6 or WG-SS in the analytic sample for the regression. We used a linear probability model in our primary specification for ease of interpretation of the coefficients, however the results are robust to an alternative specification using a logistic regression (See Table A1).

## 3. Results

**Fig. 1.**
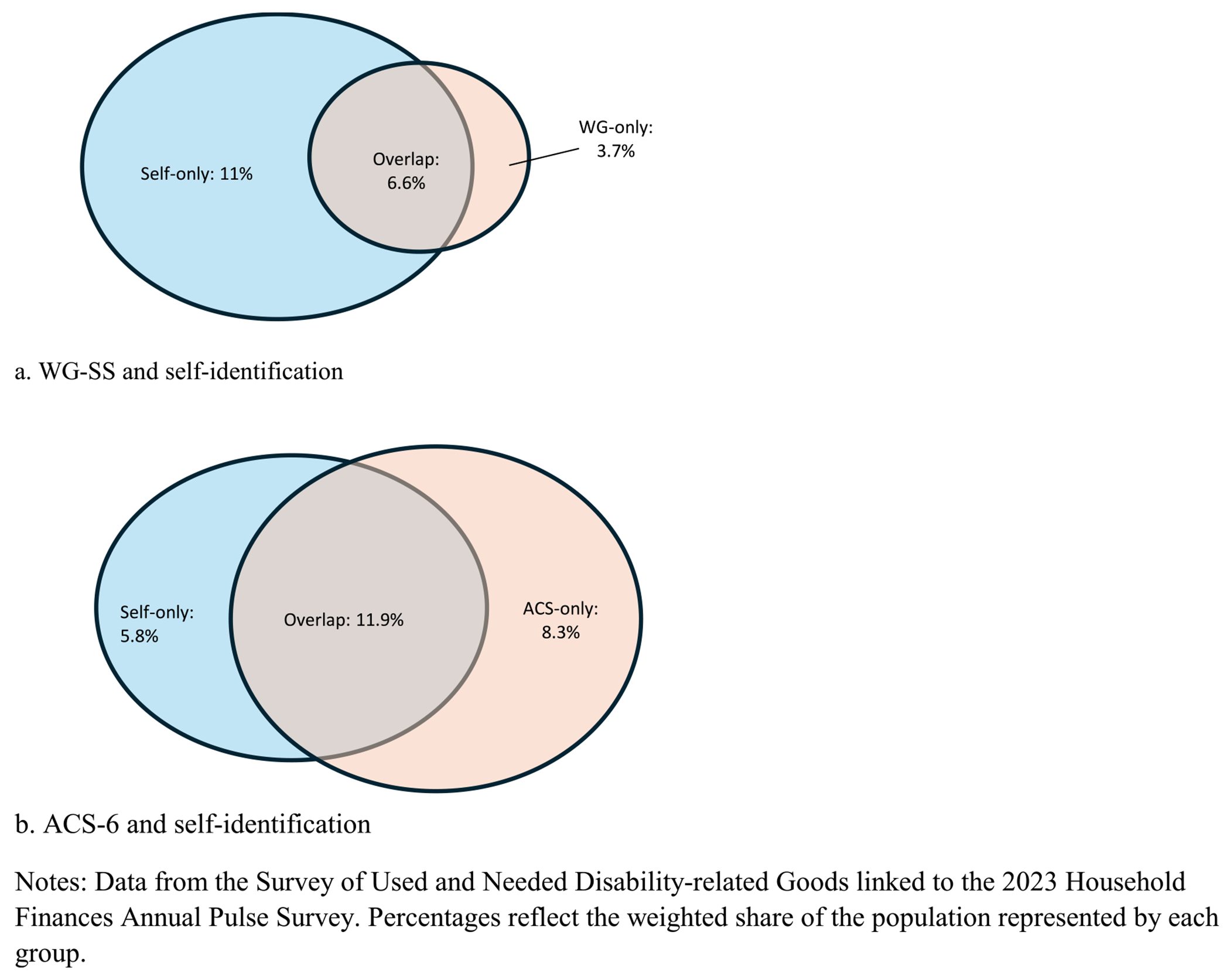
Population prevalence captured by functional questions and self-identification NOTES: Percentages reflect the weighted share of the population represented by each group. SOURCE: Survey of Used and Needed Disability-related Goods linked to the 2023 Financial Health Pulse Survey.

Fig. 1 presents two Venn-diagrams showing the prevalence estimates from comparisons between a self-identification question and the WG-SS or ACS-6. The diagrams present the extent of overlap between self-identification and functional measures (hereafter, the “overlap group”); the population who self-identifies with a disability but is not captured by functional measures (the “ID-only group”); and the population identified with disabilities via functional measures but not via self-identification (the “functional-only, WG-only or ACS-only group”). Prior research has shown that these three areas do not perfectly overlap^3,7,8,14^; the relative size of each circle, and extent of overlap between them, remain under debate.

In our survey, the WG-SS identifies approximately 10 % of the adult population as having a disability (the small orange circle in Fig. 1a). Approximately 6.6 %, or two-thirds of the WG-SS population, is also identified via self-identification. However, adding the self-identification question captures an additional 11 % of the population who are not captured by WG-SS. Combining a self-identification question and the WG-SS approximately doubles the population with disabilities to 21 % relative to the size identified by the WG-SS only (10 %). The ACS, on the other hand, identifies nearly 20 % of the population as having a disability (Fig. 1b). This finding is consistent with prior work showing that the ACS-6 identifies a population with disabilities about twice as large as the population identified by the WG-SS.^11^ Over half of the ACS-6 group - 11.9 % of the total population - also self-identifies with a disability. An additional 5.8 % of the population self-identifies but is not captured by ACS-6. Using both the ACS-6 and self-identification question increases the total population with a disability to 26 %, an increase of 30 % relative to the population captured by ACS-6 only.

Table 1 compares demographic and health characteristics between the overlap, ID-only and functional-only groups. Panel A shows the comparison between self-identification and WG-SS while Panel B shows the comparison between self-identification and ACS-6. In comparisons with both functional question series, the overlap group is older (average age 51–52), has the lowest incomes (88–89 % with less than median household income), is most likely to report having a work-limiting disability (57–60 %), being troubled with pain (75–77 %), and has the highest rates of anxiety and depression (15–20 %).

The ID-only group is significantly less likely to be working and significantly more likely to report a work-limiting disability and receipt of Social Security Disability benefits when compared to both functional-only groups (32 % vs 62 % in Panel A; 39 % vs. 60 % in Panel B). The ID-only groups are also less likely to be female and less likely to identify as Hispanic or Latino than the functional-only groups. While the ID-only group is significantly more likely to often be troubled by pain than the WG-only group (57 % vs. 40 %), there is no significant difference in rates of pain between the ID-only and ACS-only groups. By contrast, the ACS-only group is marginally significantly more likely to report anxiety and depression when compared to the ID-only group.

We also investigated the health characteristics of the ID-only and functional-only groups to understand what groups may be missed by one measure or another. Table 1 shows that 76–85 % of those ID-only group report “some” difficulty with at least one functional domain and 45–63 % of the ID-only group report “some” difficulty with two functional domains. On the other hand, 14–22 % of the ID-only group report no difficulty in all six functional domains. Figure A2 shows the distribution of WG responses among the ID-only, ACS and overlap groups for further detail on the overlap between the two question sets.

In Table 2, Panel A shows that the two most common self-reported impairments in the ID-only group are physical disabilities (52–59 %), and mental disabilities (22–29 %). The ranking of common impairments is the same when contrasting the ID-only group against those included in either WG-SS or ACS-6. In Panel B, we compare reported difficulties for the functional-only groups who do not self identify with a disability. While the question wording differs depending on the comparison with WG-SS and ACS, we organize responses based on the functional domain covered by the questions. The relative ranking of impairments represented in the functional-only groups are again quite similar. The most common functional difficulty is difficulty remembering, indicated among 47 % of the WG-SS-only group and 43 % of the ACS-only group. Sensory difficulties (hearing or seeing) are the next two most common functional impairments. Self-care was the least commonly reported difficulty in both groups.

**Fig. 2.**
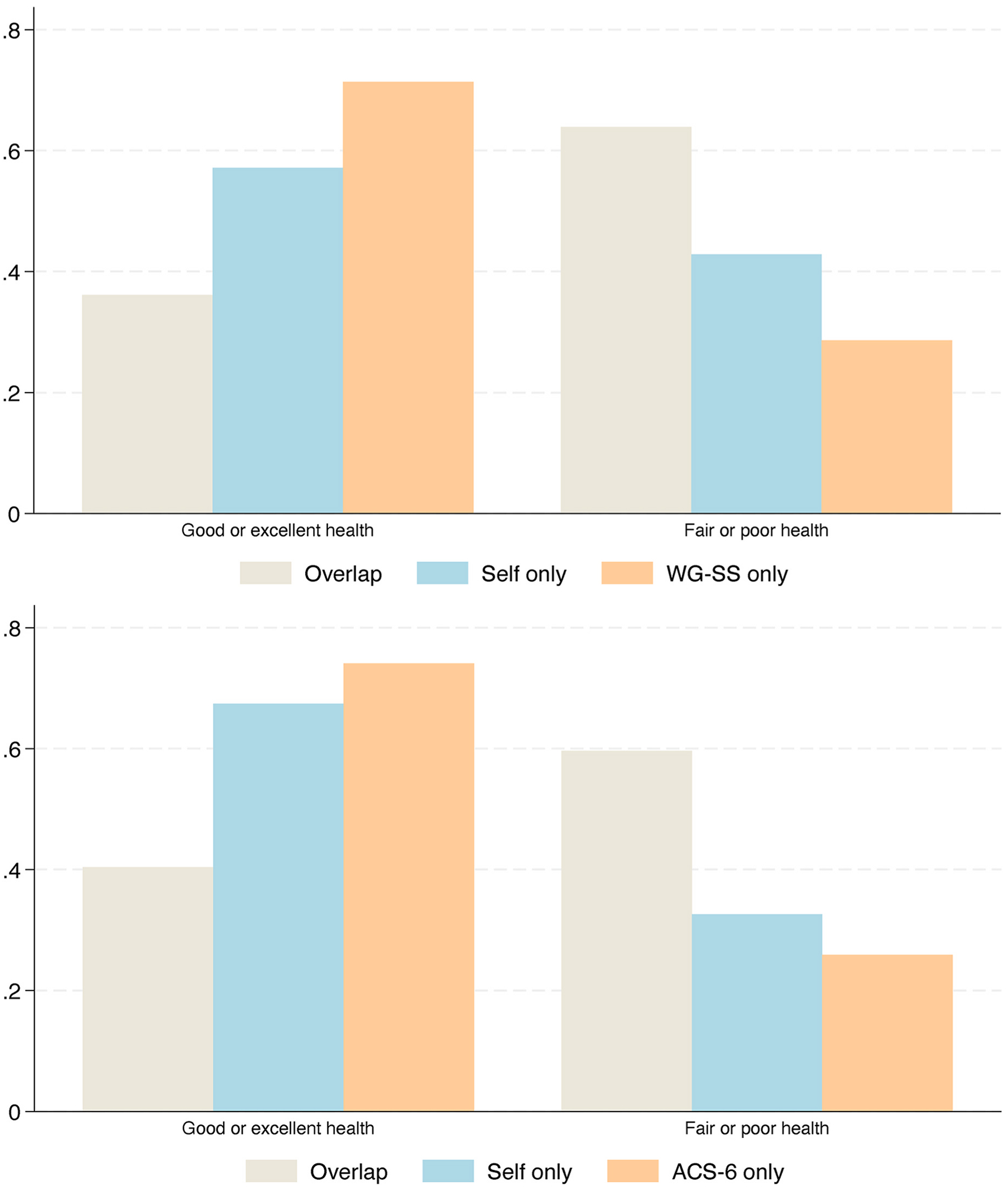
Distribution of Self-Reported Health among individuals in the Overlap, ID-only and Functioning-only groups (a) Self identification vs. WG-SS (b) Self identification vs ACS-6 NOTES: Figures show the distribution of self-reported health across the overlapping group, self-ID only and functioning only comparisons for both the WG-SS and ACS-6 comparisons. Chi-squared test revealed that differences in good or excellent and fair or poor health between groups were statistically significant at the 5 % level. SOURCE: Survey of Used and Needed Disability-related Goods linked to the 2023 Financial Health Pulse Survey.

Fig. 2 further compares self-reported health between these three groups. Comparisons of the ID-only group with the ACS and WG-SS groups again reveal similar patterns. Approximately 60 % of the overlap group based on either functioning series report being in fair or poor health. Forty three percent and 28 % of the ID-only groups report fair or poor health in the WG-SS and ACS comparisons, respectively. The functional-only groups report fair or poor health at the lowest rates. The comparison of self-reported mental health shows the same gradient in the comparison with the WG-SS (see Appendix Figure S2). However, when compared to the ACS-only group, the ID-only group reports slightly better mental health, consistent with the findings on anxiety and depression in Table 1.

**Fig. 3.**
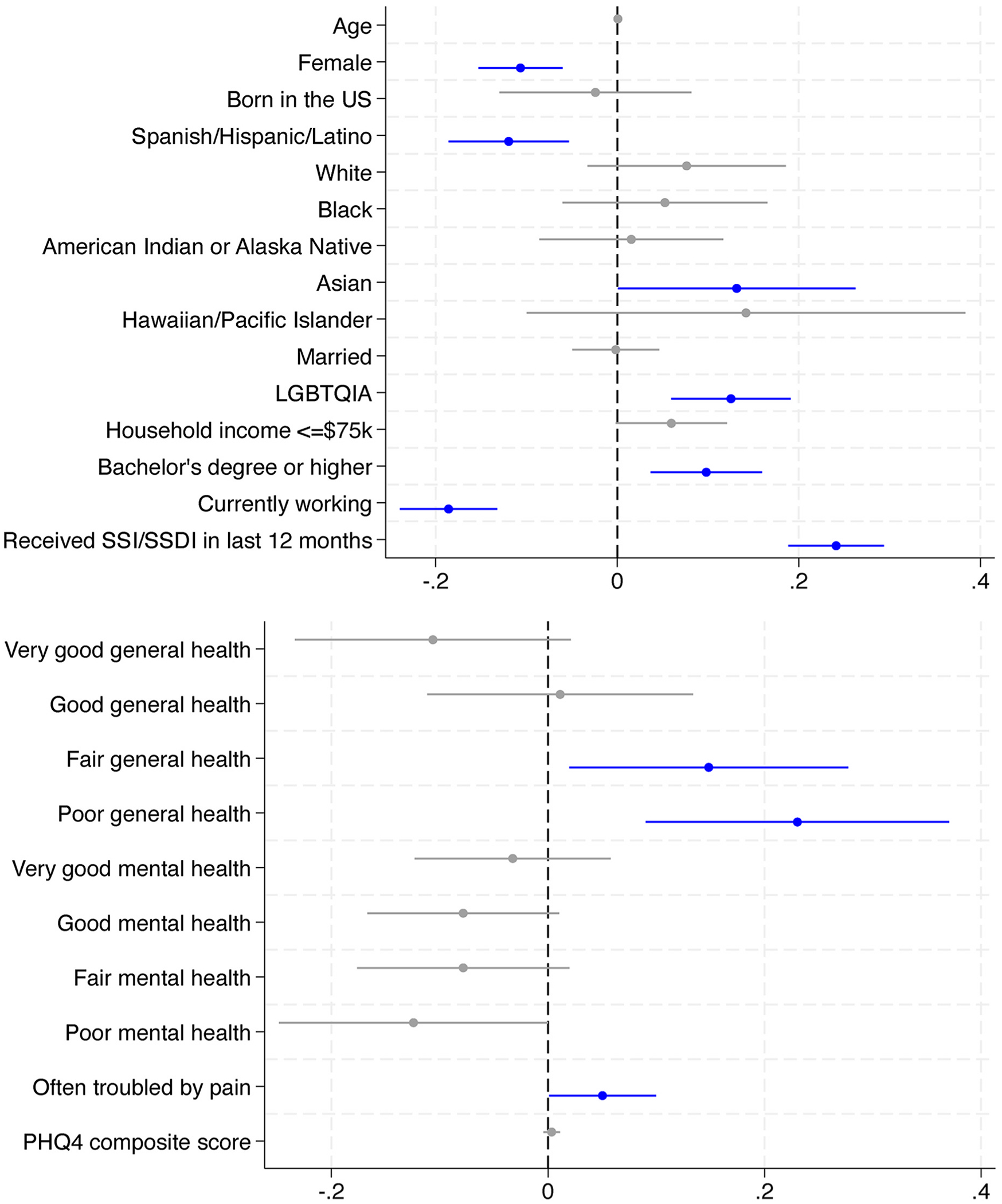
Coefficients from multivariable regression predicting self-identification (a) Demographic characteristics (b) Health Characteristics NOTES: Figures show the coefficients from a linear probability model regressing and indicator for self identification on all of the demographic and health characteristics shown above. Figures a and b show coefficients from the same model. Coefficients shown in blue are statistically significant at the 5 % level. (For interpretation of the references to colour in this figure legend, the reader is referred to the Web version of this article.) SOURCE: Survey of Used and Needed Disability-related Goods linked to the 2023 Financial Health Pulse Survey.

Fig. 3 presents coefficients from the multivariable regression exploring which demographic and health characteristics are predictive of the dependent variable of self-identifying with a disability (see Table A1 for corresponding coefficients). Several patterns from Table 1 and Fig. 2 persist in this regression: those who identify as female, Hispanic/Latinx, or who are currently working are significantly less likely to self-identify as having a disability compared to respondents in the functional-only groups. Those who participate in disability programs, identify as LGBTQIA+, have higher levels of education (Bachelor’s degree or higher), and who are Asian are more likely to self-identify than those in the functional-only groups. Individuals in fair or poor self-reported physical health are more likely to self-identify, as well as those who report being in pain often.^d^

Next, we examine expenditures and unmet needs for disability-related goods and services for respondents who self-identified or were screened in by the WG-SS, using the sample of SDGS respondents. Table 3 shows that those in the overlap group spend the largest amount out-of-pocket on disability related costs (approximately $6600 in $2023) and have the highest reported unmet needs. The out-of-pocket disability-related costs between the ID-only and WG-only groups are not significantly different, but the ID-only group reports higher unmet needs in several domains including mobility (15 % vs. 3 %) between ID-only and WG-SS only and general goods not covered by other domains in table (22 % vs. 15 %). The WG-SS only group reports higher unmet needs for hearing-related items (23 % vs. 12 %).

## 4. Discussion

We compare the populations with disabilities identified through functional questions and a self-identification question to understand potential advantages and drawbacks of including a self-identification question in surveys. The results lead to two main conclusions. First, adding a self-identification question will also expand the size of the population with disabilities, though the extent of this expansion varies when comparing to the WG-SS (doubling the size of the population) versus ACS (increasing by 30 %). Second, adding a self-identification question in addition to functional questions could help target a population with the worst health and highest unmet needs within the population with disabilities.

The comparisons with both WG-SS and ACS together provide a consistent picture of the characteristics of those included under various parts of the Venn diagram shown in Fig. 1. First, the overlap group is in significantly worse physical and mental health, report higher rates of pain, depression and anxiety, have the highest financial constraints (e. g., lower household incomes, higher out-of-pocket disability-related costs and disability related unmet needs) and highest disability program participation. Adding a self-identification question could thus be an effective way to identify individuals most in need of services and support within the population identified by functional questions. This point is consistent with the conclusions of Hall et al., 2024 with respect to ACS-6,^14^ but has not been analyzed with respect to WG-SS.

Those who are in the ID-only group seem to fall in the middle: they are in worse self-reported health than those in the functional-only group significantly less likely to be working, and more likely to have a work-limiting disability. Those in the ID-only group are most likely to have a physical or mental disability and report higher rates of unmet need for mobility-related items than the functional-only group. Relative to the WG-only group, the ID-only group also reports significantly higher rates of pain. A majority of the ID-only group also reports “some” difficulty with multiple functional domains based on the WG-SS questions. Together, these findings suggest that the ID-only could include those with physical impairments which are not severe enough to be captured by the WG-SS, or don’t involve difficulty walking (the main physical question asked in functioning questions). This group could include those with other difficulties such as back problems or chronic fatigue which may be missed by the wording of functional questions. Indeed, 14 % of the ID-only group in the WG-SS comparison and 22 % of the ACS comparison reported “no” difficulty with all of the functional domains. The ID-only population could also include those with physical conditions which intermittently cause impairment, or which are accommodated to some degree, resulting in the respondents answering “no” on the functional question but nevertheless self-identifying as having a disability. Those captured in the functional-only group are most likely to have difficulty concentrating or remembering or sensory difficulties. While these impairments could be common issues of aging, the functional-only groups are not significantly older than the ID-only group, and in fact the WG-only group is significantly younger than the ID-only group.

The main difference between the comparison with WG-SS and ACS is the relative size of these populations. Two-thirds of the self-identified population is not captured by the WG-SS, compared to one-third not captured by ACS-6. This difference results in part from the fact that the ACS-6 identifies a larger population to begin with: the size of the ACS circle in the Venn diagram is larger, so to speak, making it easier to have overlap with the self-identified population. However, it means that adding a self-identification question to surveys will have different implications for changing the overall prevalence depending on what functional questions were previously included in the survey: adding a self-identification question will increase prevalence to a greater extent in surveys that previously only asked the WG-SS.

Despite the rich information we can analyze from this novel data source, there are some limitations to our findings. We lack detailed health information which would enable us to further explore certain groups which could be under identified by functional screeners, including those with psychological or behavioral health impairments, or those with chronic fatigue. Our survey also does not address the chronicity or frequency with which impairments affect respondents. Furthermore, some limitations arise from the online format of the survey. First, some groups of individuals with disabilities are likely under-represented if they have difficulties using the internet - for example, individuals with intellectual disabilities. More generally, there are well documented differences in internet access across geography (urban vs. rural areas), socioeconomic characteristics and cultural beliefs and preferences.^18,19^ While the UAS survey weights adjust for these observable differences to match nationally representative population targets, there nevertheless could be remaining bias in the sample due to unobservable differences in the populations who opt in to participation in online surveys.^20^ Finally, the question about self-identification in the survey uses person-first language. Several different surveys have different wording questions regarding disability questions,^7,8^ and all have potential drawbacks of excluding some respondents. In our case, individuals who prefer identify-first language could be less likely to endorse a self-identification question using person-first language,^21^ resulting in this group being under-represented in our analysis.

This analysis highlights the complexity and nuance of identifying a population with disabilities. Disability depends on the context and environment and may have different meanings depending on a person’s identity and cultural background. It is also measured for different purposes in surveys including: to estimate the size of the population for representation purposes, to identify populations in need of social supports, and to examine disparities relative to other groups. As a result, the right disability measure likely also depends on the context and objective. If the intention is to primarily identify the size of the population for representation purposes, then the most expansive definition, including those captured by either functional questions or a self-identification question may be most appropriate. However, our analysis also revealed those endorsed by *both* the self-identification question and a functional measures - the most stringent definition - identifies a population with the highest functional and financial needs. If the purpose is to identify individuals at the highest risk from, for example, a disaster event, then identifying who self-identify *and* are captured by functional measures may be the most useful. The inclusion of a self-identification measure thus could allow researchers and policymakers to use one or both constructs to identify the population depending on their circumstance. Ultimately, there is no one “perfect” measure for identifying populations with a disability, and the choice of which measure to use should take into consideration the tradeoffs of individuals who would be included or excluded depending on the measure, as highlighted in this analysis.

## 5. Conclusion

The population of people with disabilities is diverse in many ways, making it difficult to distill measurement down into one question or set of questions to be administered on public surveys. In this paper we consider the role that self-identification may play in this measurement challenge. Our findings suggest that including a self-identification question may be one piece to the larger puzzle but likely would not capture the entire population of people with disabilities if used to replace a functional screener. Complex topics require comprehensive solutions, and the challenge of measuring disability will likely be best served by an approach which includes a functional screener in addition to a self-identification question to capture the largest population and include the most flexible options for measurement purposes for public programs.

## Supplementary Material

1

2

3

4

5

6

7

8

## Figures and Tables

**Table 1:** Demographic and health characteristics of subgroups in comparison of functioning questions and self-identification question.

	Panel A: WGSS vs SelfID				Panel B: ACS-6 vs SelfID			
	(1)	(2)	(3)	(4)	(1)	(2)	(3)	(4)
	Overlap	ID-only	WG-only	P-value (Col 2 vs. 3)	Overlap	ID-only	ACS-only	P-value (Col 2 vs. 3)
% of self-identified population (wt)	38 %	62 %			69 %	31 %		
*Demographics*								
Age	52.53	48.10	41.16	0.00	51.31	46.59	45.91	0.94
Female	0.60	0.50	0.70	0.00	0.57	0.46	0.61	0.00
Hispanic or Latino	0.12	0.12	0.24	0.04	0.13	0.11	0.25	0.00
White	0.80	0.77	0.75	0.25	0.78	0.79	0.81	0.24
Black	0.17	0.20	0.21	0.17	0.19	0.17	0.15	0.18
Married	0.39	0.43	0.47	0.91	0.39	0.47	0.47	0.96
LGBTQIA+	0.18	0.16	0.16	0.65	0.16	0.18	0.13	0.14
Household income<$75k	0.89	0.79	0.71	0.10	0.88	0.73	0.71	0.38
BA or higher	0.11	0.22	0.21	0.34	0.14	0.24	0.22	0.44
Currently working	0.20	0.32	0.62	0.00	0.22	0.39	0.60	0.00
Work limiting disability	0.60	0.45	0.05	0.00	0.57	0.37	0.04	0.00
Social Security disability benefits	0.47	0.42	0.04	0.00	0.47	0.39	0.02	0.00
*Health Measures*								
Often troubled by pain	0.77	0.57	0.40	0.01	0.75	0.45	0.51	0.13
PHQ4 composite score	4.03	3.22	2.87	0.62	4.06	2.43	2.99	0.04
WG-SS Depression	0.15	0.11	0.12	0.26	0.15	0.06	0.10	0.10
WG-SS Anxiety	0.20	0.13	0.19	0.14	0.18	0.11	0.16	0.07
Answered “no difficulty” to all WG questions	–	0.14	–	0.00	0.02	0.22	0.15	0.07

Answered “no” to all ACS questions	0.09	0.47	0.58		–	1.00	–	

*Number of WG-SS functional domains with “some difficulty”*								

0	0.09	0.14	0.21	0.37	0.06	0.24	0.17	0.07
1	0.27	0.23	0.33	0.88	0.21	0.31	0.30	0.58
2	0.33	0.28	0.29	0.39	0.32	0.24	0.31	0.03
3	0.18	0.18	0.13	0.38	0.21	0.11	0.15	0.23
4	0.10	0.12	0.04	0.14	0.13	0.07	0.05	0.09
5	0.04	0.05	0.00	0.13	0.06	0.02	0.02	0.82
Observations (unwt)	372	608	236		658	322	517	

NOTES: Panel A compares the overlap between a disability self-identification question with the WG-SS functional questions. Panel B compares the overlap between the disability self-identification question with the ACS-6. Sample proportions are presented for binary variables. Depression and Anxiety were assessed using questions in the broader Washington Group question series on these topics. Individuals were indicated as having depression or anxiety if they answered that they experienced depression or anxiety daily and that the level of feelings was “a lot”. Sample sizes reflect the total number of survey respondents identified as having a disability in each group. Statistics calculated with survey weights.

SOURCE: Survey of Used and Needed Disability-related Goods linked to the 2023 Financial Health Pulse Survey.

**Table 2:** Health impairments of non-overlapping groups.

Panel A: Self reported disabilities, Self-ID only		
	(1)	(2)
	WG-SS comparison	ACS comparison
Physical disability	0.59	0.52
Mental disability	0.29	0.22
Other disability	0.12	0.14
Learning disability	0.11	0.11
Vision disability	0.12	0.09
Hearing disability	0.06	0.03
Developmental disability	0.04	0.05
Intellectual disability	0.02	0.02
Identified under ACS	0.53	–
Identified under WG-SS	–	0.19

Observations (unwt)	608	322
Panel B: Functional limitations, Functioning only groups		
	(1)	(2)
	WG-SS only	ACS only

Remembering	0.47	0.43
Seeing	0.31	0.28
Hearing	0.17	0.21
Walking	0.13	0.20
Communicating/errands*	0.05	0.13
Self-care	0.03	0.02

Observations (unwt)	236	517

NOTES: Panel A presents the proportions self-reported disabilities for the group who self-identified as having a disability but did not screen as having a disability based on a functional screener. Column 1 presents the characteristics of the group only self-identified with a disability in the comparison with WG-SS, and Column 2 presents the characteristics for the group only self-identified in the comparison with ACS-6. Panel B presents the proportions of functional limitations of those who screened as having a disability based on the functional screener listed in the column header, but who did not self-identify. For each respective group, the shares reflect the share meeting the screening criteria for the question related to the functional domain in the row. For example, the first column show the share of the WG-SS only group who reported “a lot of difficulty” or “cannot do at all” to the question about difficulty remembering, while the second column reports the share of the ACS-only group who reported “yes” to the question about difficulty remembering. One functional domain differs between WG-SS and ACS: the WG-SS asks about difficulty communicating, while the ACS-6 asks about difficulty running errands. We show the shares reporting difficulty with this domain on the same row, with the corresponding numbers for WG-SS corresponding to difficulty communicating, and the numbers for ACS-6 corresponding to difficulty running errands.

SOURCE: Survey of Used and Needed Disability-related Goods linked to the 2023 Financial Health Pulse Survey.

**Table 3:** Out-of-pocket disability related costs and unmet needs for disability-related goods.

	(1)	(2)	(3)	(4)
	Self + WG-SS	Self only	WG-only	P-value (Col 2 vs. 3)
Total OOP costs	6593.07	5609.72	6107.04	0.73
Any unmet need	0.79	0.65	0.60	0.20
Any unmet need for mobility items	0.38	0.15	0.03	0.00
Any unmet need for assistive technologies	0.32	0.14	0.12	0.42
Any unmet need for external home modifications	0.22	0.09	0.08	0.52
Any unmet need for general goods	0.30	0.22	0.15	0.02
Any unmet need for health services	0.43	0.30	0.27	0.30
Any unmet need for health goods	0.31	0.24	0.20	0.22
Any unmet need for hearing items	0.14	0.12	0.23	0.00
Any unmet need for internal home modifications	0.24	0.10	0.06	0.07

Observations (unwt)	410	717	272	

NOTES: Analysis conducted with survey weights. Table shows average OOP costs and proportion of the column sample with unmet needs. Questions about unmet needs and out-of-pocket costs and unmet needs for disability-related goods were not asked in the Financial Health Pulse Survey, so this table is not shown for comparisons with the ACS-6.

SOURCE: Survey of Used and Needed Disability-Related Goods, 2023

## Footnotes

c We note that the extended WG question series does include questions about anxiety and depression, though these questions are not included in the short set.

d Table A1 shows similar results with a dependent variable of an indicator for being in the overlap group. Again consistent with Table 1 and Fig. 2, these individuals are more likely to be older, LGBTQIA+, less likely to be working and more likely to receive disability insurance, in worse physical health, more likely to be in pain and have higher PHQ-4 scores than respondents in the ID-only or functional-only groups.

## Appendix A. Supplementary data

Supplementary data to this article can be found online at https://doi.org/10.1016/j.dhjo.2025.101980.
