# Supplementary material for: A comparative analysis of self-identification and functional measures of disability
Source: Disabil Health J. Author manuscript; Available in PMC 2026 Jul 15. (PMC13371818; doi:10.1016/j.dhjo.2025.101980)
Supplement: 1 [file NIHMS2185896-supplement-1.docx]

**Figure S1: Consort Diagram of Sample Selection**
