# Supplementary figures and images for: A comparative analysis of self-identification and functional measures of disability
Source: Disabil Health J. Author manuscript; Available in PMC 2026 Jul 15. (PMC13371818; doi:10.1016/j.dhjo.2025.101980)

Distribution of WG-SS scale for difficulty walking

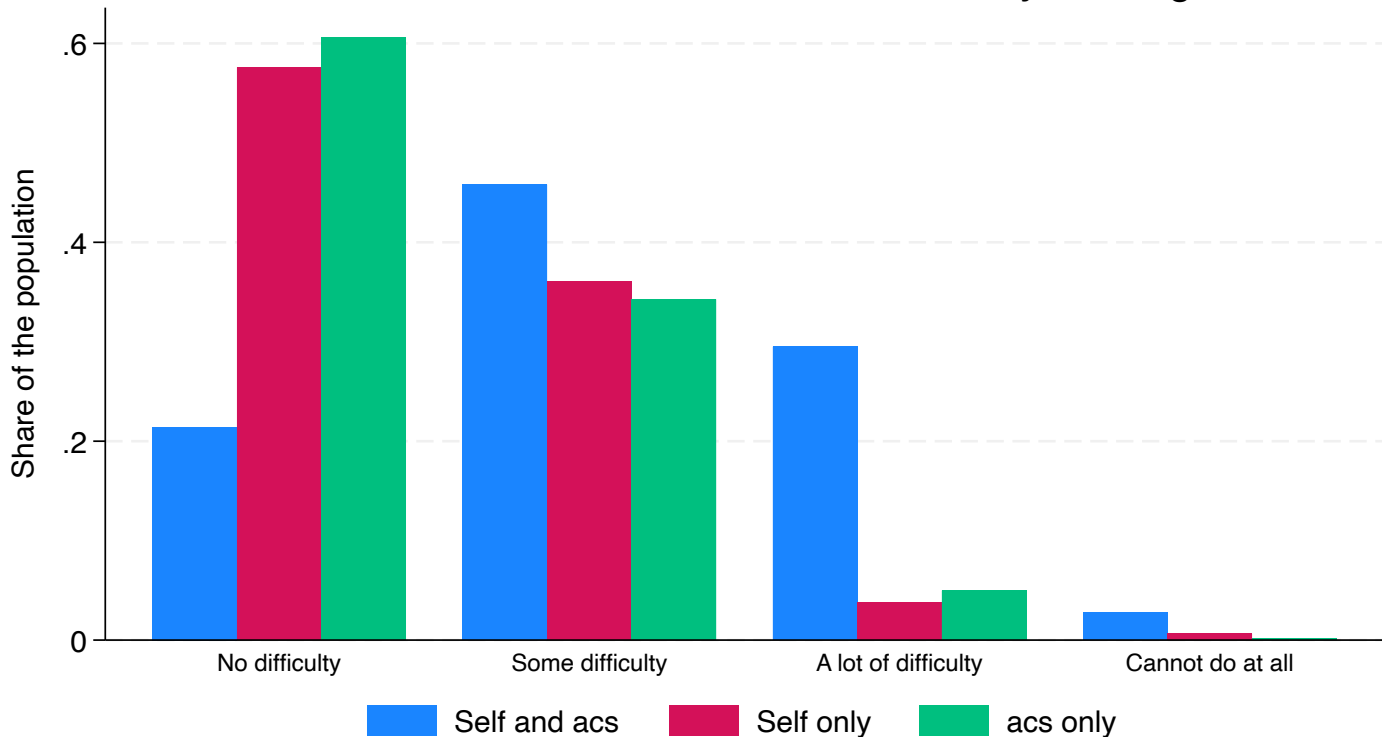

Supplement: 2 [file NIHMS2185896-supplement-2.pdf]

# Distribution of WG-SS scale for difficulty seeing

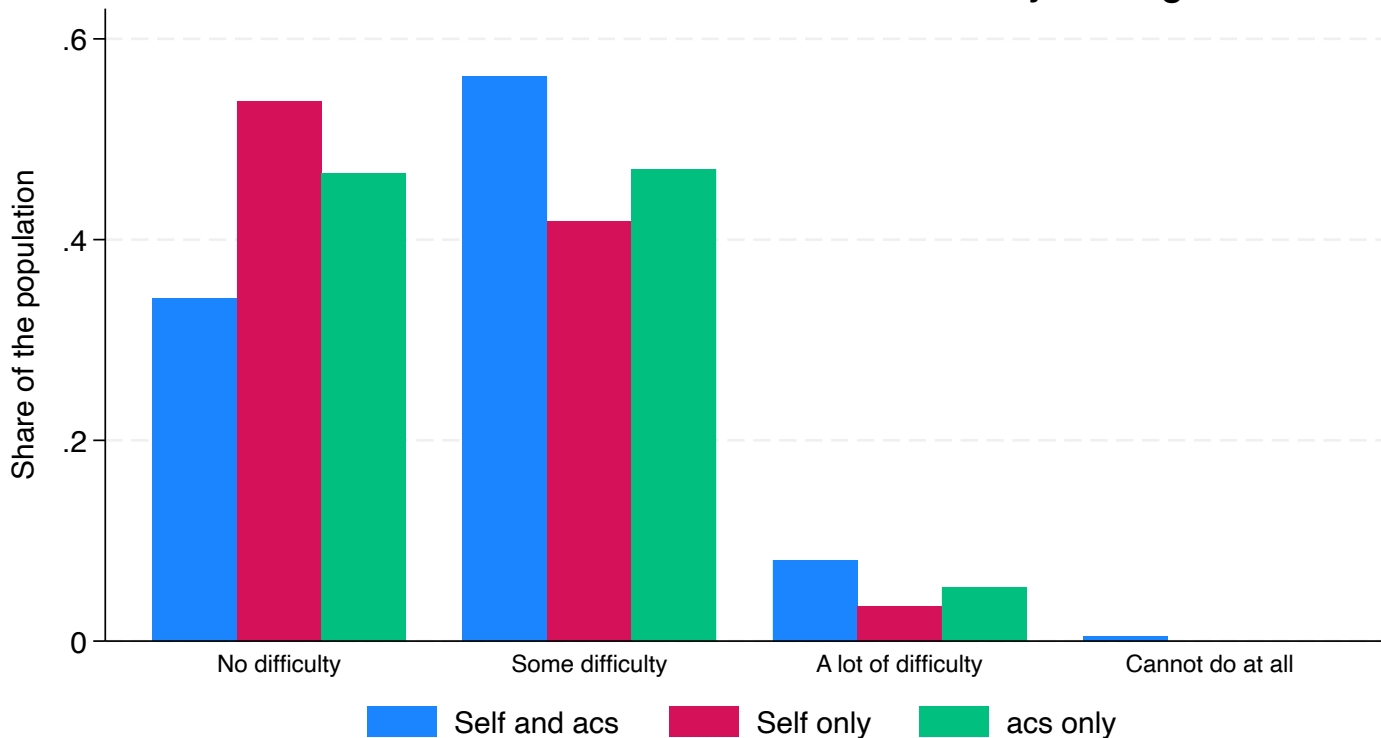

Supplement: 3 [file NIHMS2185896-supplement-3.pdf]

# Distribution of WG-SS scale for difficulty hearing

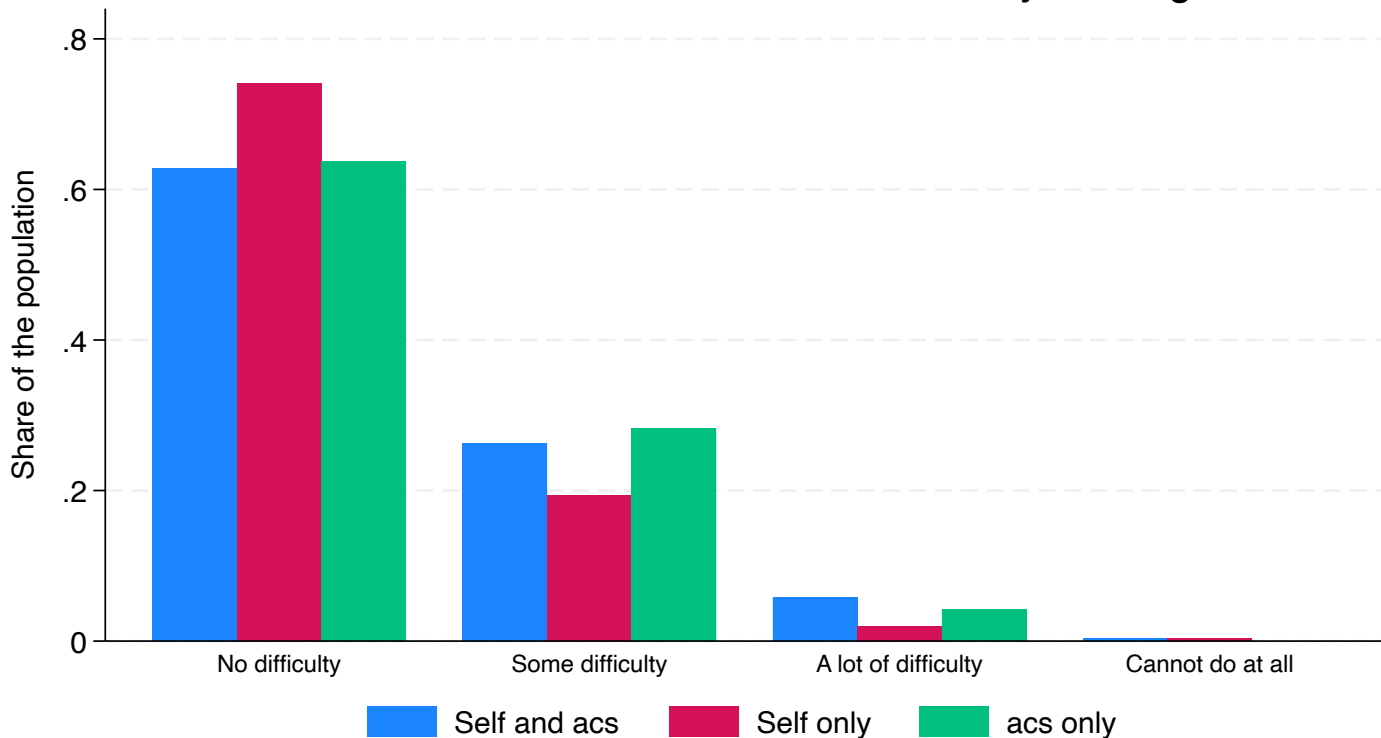

Supplement: 4 [file NIHMS2185896-supplement-4.pdf]

# Distribution of WG-SS scale for difficulty self care

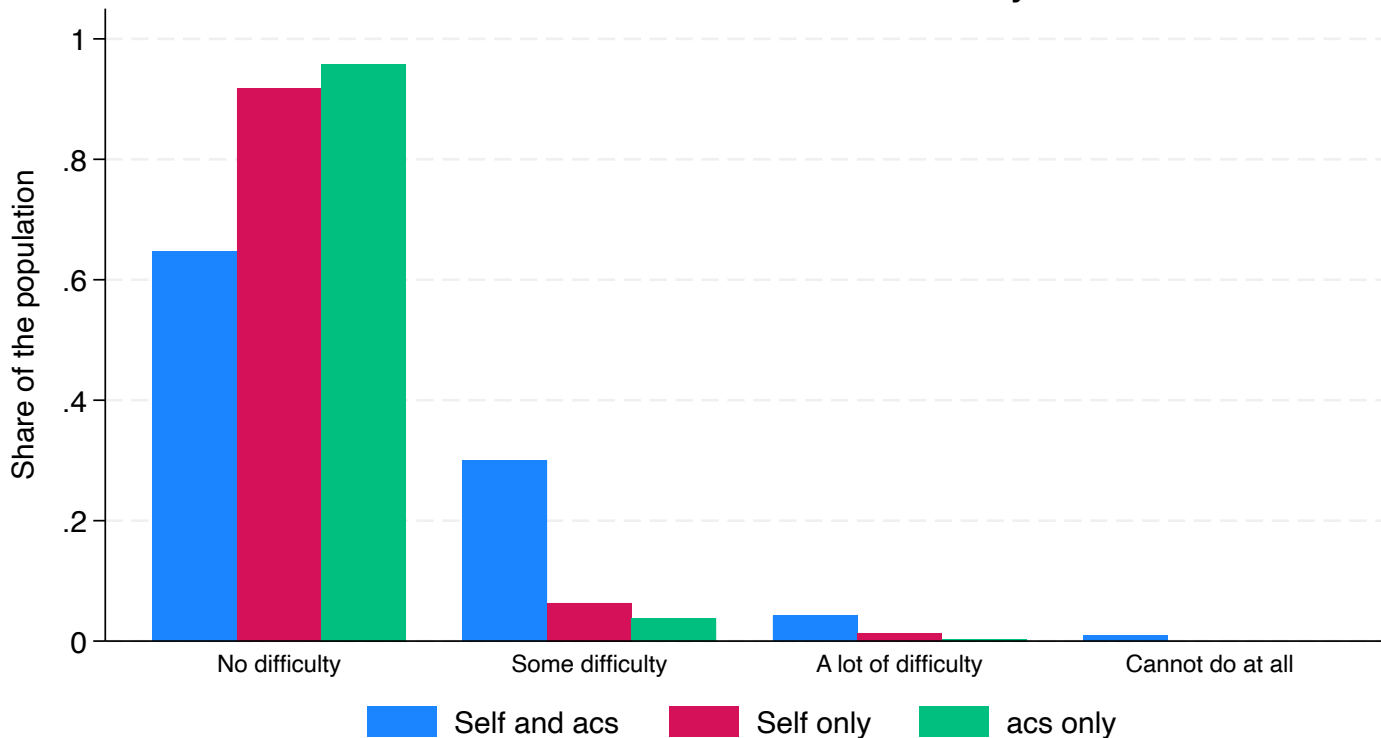

Supplement: 5 [file NIHMS2185896-supplement-5.pdf]

# Distribution of WG-SS scale for difficulty remembering

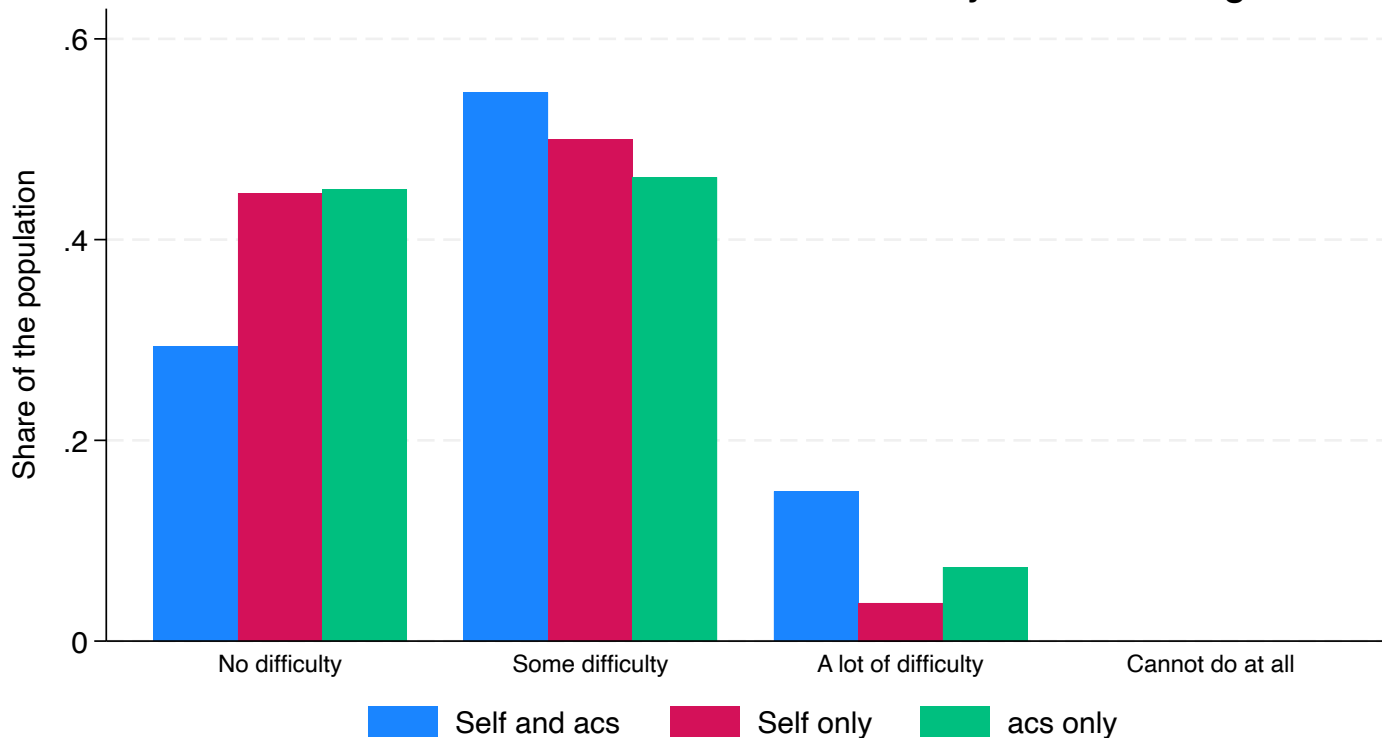

Supplement: 6 [file NIHMS2185896-supplement-6.pdf]

Distribution of mental health, by WG-SS group

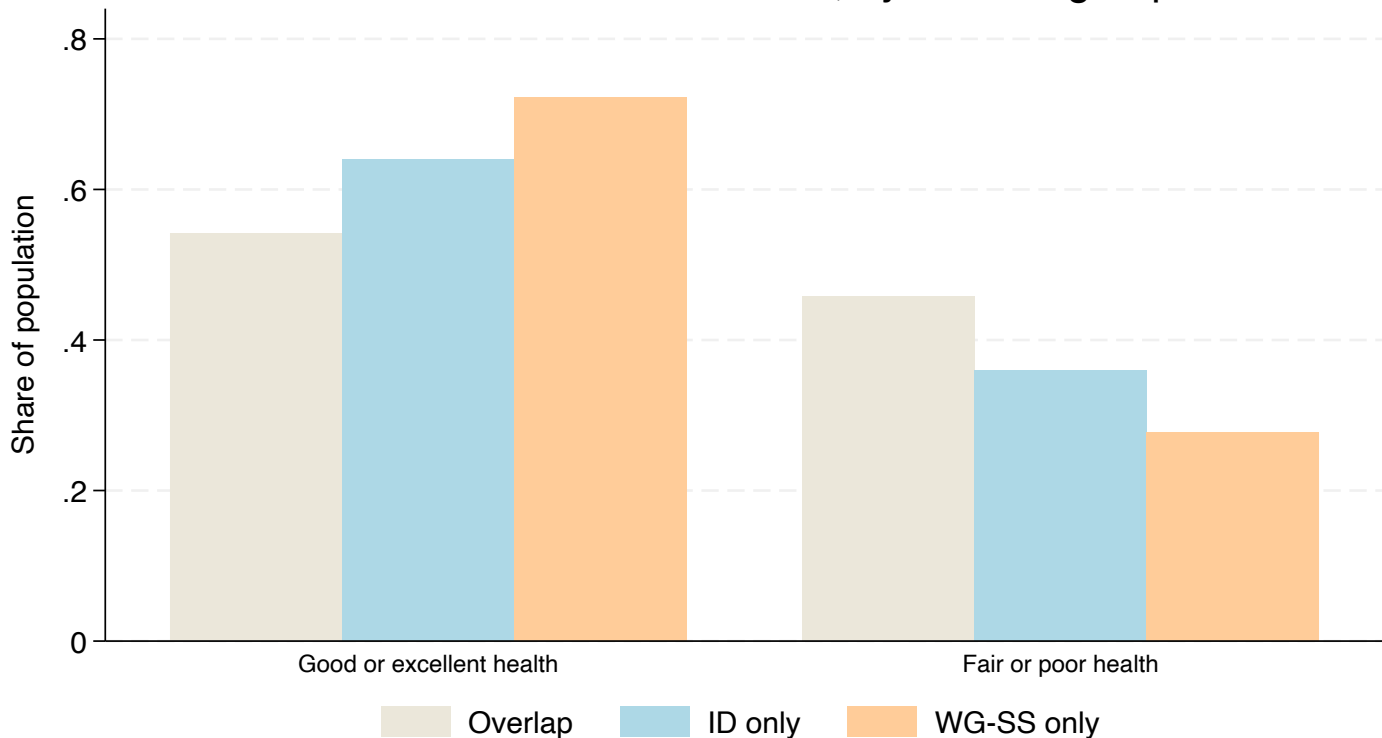

Supplement: 7 [file NIHMS2185896-supplement-7.pdf]

Distribution of mental health, by ACS-6 group

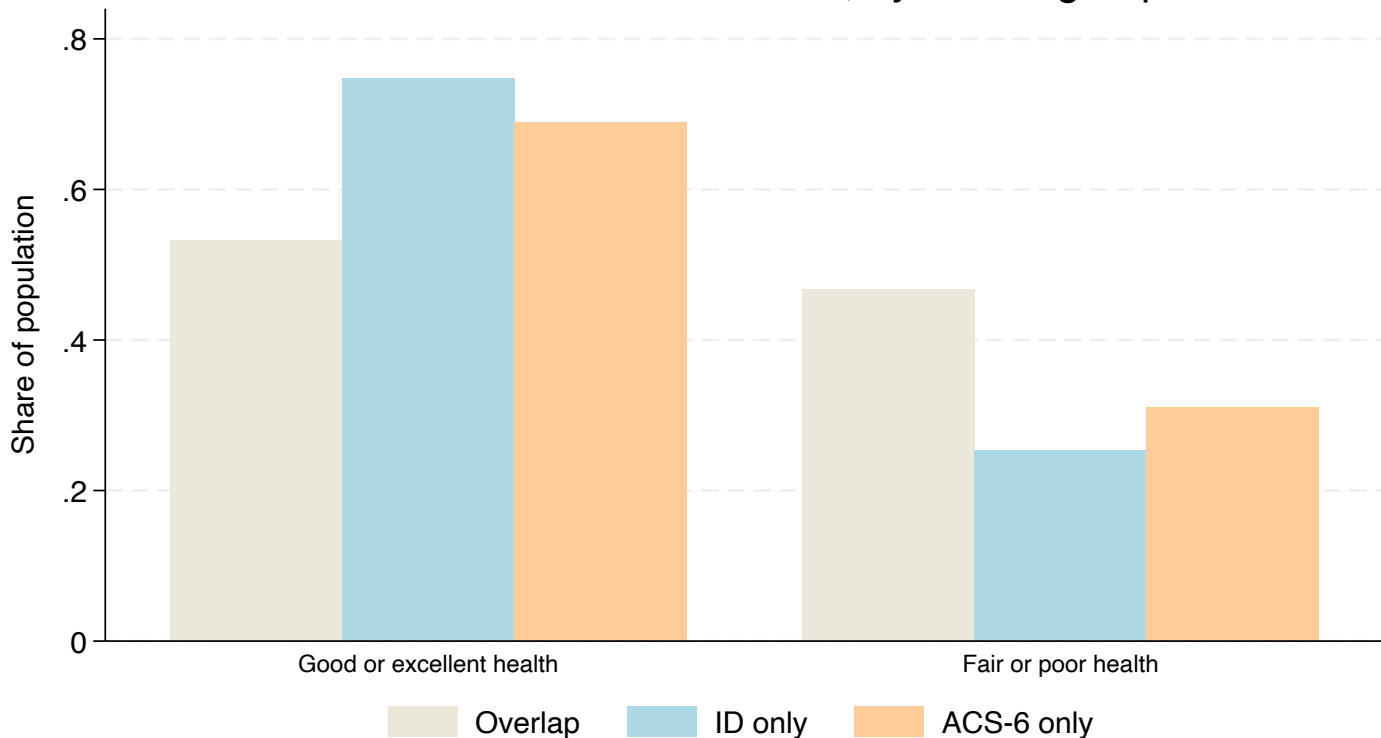

Supplement: 8 [file NIHMS2185896-supplement-8.pdf]
